# Deciphering the Genetic Complexity of Classical Hodgkin Lymphoma: Insights and Effective Strategies

**DOI:** 10.2174/0113892029301904240513045755

**Published:** 2024-05-24

**Authors:** Chaeyoung Lee, Yeeun An

**Affiliations:** 1 Department of Bioinformatics and Life Science, Soongsil University, Seoul 06978, Korea

**Keywords:** Hodgkin lymphoma, hodgkin and reed-sternberg cell, expression quantitative trait locus, genetic etiology, genetic heterogeneity, genetic lesion, genome-wide association study, NF-kB

## Abstract

Understanding the genetics of susceptibility to classical Hodgkin lymphoma (cHL) is considerably limited compared to other cancers due to the rare Hodgkin and Reed-Sternberg (HRS) tumor cells, which coexist with the predominant non-malignant microenvironment. This article offers insights into genetic abnormalities in cHL, as well as nucleotide variants and their associated target genes, elucidated through recent technological advancements. Oncogenomes in HRS cells highlight the survival and proliferation of these cells through hyperactive signaling in specific pathways (*e.g*., NF-kB) and their interplay with microenvironmental cells (*e.g*., CD4+ T cells). In contrast, the susceptibility genes identified from genome-wide association studies and expression quantitative trait locus analyses only vaguely implicate their potential roles in susceptibility to more general cancers. To pave the way for the era of precision oncology, more intensive efforts are imperative, employing the following strategies: exploring genetic heterogeneity by gender and cHL subtype, investigating colocalization with various types of expression quantitative trait loci, and leveraging single-cell analysis. These approaches provide valuable perspectives for unraveling the genetic complexities of cHL.

## 1 INTRODUCTION

Classical Hodgkin lymphoma (cHL) is characterized by the presence of malignant Hodgkin and Reed-Sternberg (HRS) tumor cells, which include both mononuclear Hodgkin cells and multinuclear Reed-Sternberg cells resulting from cytokinesis failure. These cells originate from impaired germinal center B lymphocytes with genomic rearrangements and hypermutations occurring in variable, diversity, and joining genes encoding immunoglobulins and T-cell receptors during pre-malignant B cell development [1, 2]. However, the exact cause of these mutations and the etiology of cHL remain unclear.

The genetics of cHL have drawn attention due to its increased risk in individuals with a family history of the disease. For instance, the concordance rate (5.6% = 10/179) between monozygotic twins was higher than that (0.0% = 0/187) between dizygotic twins [3], and the risk for cHL was increased 3.3-fold in first-degree relatives of Nordic patients [4] and 5.4-fold in French children with a family history [5]. Nevertheless, genetic studies of cHL have lagged behind other cancer types due to the rarity of HRS tumor cells within a complex microenvironment. Recent technological advancements have improved our understanding of genetic aberrations in HRS cells, and genome-wide association studies (GWAS) have identified genetic associations with cHL susceptibility, although with some limitations in resolution. This article aims to explore genetic lesions in cHL and investigate genetic variants and their associated target genes from a genetic etiology perspective.

## 2 GENETIC LESIONS IN CLASSICAL HODGKIN LYMPHOMA

Given that HRS cells typically comprise only 1% of malignant tissue in cHL [6], their detecting genetic alterations is challenging due to their intermingling with the predominant non-malignant microenvironment. However, significant progress has been made in understanding the genetic lesions that may characterize cHL, facilitated by techniques like laser microdissection, flow cytometric cell sorting, and next- generation sequencing [7, 8].

Numerous abnormal genes within HRS cells directly or indirectly suggest immunodeficiency, as illustrated in Fig. (**1**). This weakened immune state increases vulnerability to infections by viruses like Epstein-Barr virus (EBV) and Human Immunodeficiency Virus (HIV). Notably, genetic alterations in genes encoding immunoglobulins and T cell receptors, abundant in HRS cells, exemplify these abnormalities [9, 10]. Oncogenomic analyses highlight persistent activation of specific signalling pathways, as illustrated in Fig. (**1**), including nuclear factor kappa B (NF-kB), Janus kinase/signal transducer and activator of transcription (JAK/STAT), and phosphoinositide 3-kinase/protein kinase B (PI3K/AKT) pathways [8, 7, 11]. The hyperactive signalling in these pathways may contribute to the survival and proliferation of HRS cells. For instance, CD30, a hallmark in cHL, activates the NFκB pathway, supporting HRS cell survival as a tumor necrosis factor receptor superfamily member [12, 13]. Moreover, HRS cells enhance survival by engaging in a dialogue with various tumor microenvironmental cells. This interaction is facilitated by promoting the secretion of specific growth factors and cytokines *via* constitutively active signalling pathways in HRS. An illustration is the interaction of HRS cells with CD4^+^ T cells, where binding of CD40 to CD40 ligand accelerates the activated NF-kB pathway, contributing to tumor growth [14].

The microenvironment of cHL includes cytotoxic T cells and natural killer (NK) cells, although HRS cells can evade immune surveillance by these cytotoxic cells. Importantly, HRS cells commonly lack expression of MHC class I, which is crucial for recognition by cytotoxic CD8+ T cells, especially in EBV-negative cases [8, 15]. This deficiency is often due to frequent mutations in the B2M gene, essential for MHC class I assembly [16]. Additionally, HRS cells can evade cytotoxic cells by expressing surface molecules like PD-L1 and PD-L2 [17]. Intriguingly, PD-L1, known for its immunosuppressive role, can also be expressed by macrophages in the tumor microenvironment [18].

## 3 SUSCEPTIBILITY LOCI AND GENES FOR CLASSICAL HODGKIN LYMPHOMA

The pursuit of identifying genetic variants influencing susceptibility to cHL has been a major focus for geneticists, albeit with limited understanding. This is because while some of these variants act as driver mutations, contributing to cHL development and progression, many others are passenger mutations that merely accompany the disease without affecting its progression [19-21]. Genetic variants segregating within families have been identified through familial cHL studies. For example, one such study revealed the inheritance of cHL associated with a reciprocal translocation between chromosomes 2 and 3 (Table **1**) [22]. While the intergenic breakpoint was on chromosome 2, the breakpoint on chromosome 3 was located in intron 1 of KLHDC8B. Consequently, this reciprocal translocation disrupts the gene encoding KLHDC8B. KLHDC8B is crucial for cellular midbody functions, including the daughter cell connection and separation, as well as nuclear segregation during cytokinesis. Functional experiments have shown an increase in binucleated cells when KLHDC8B gene expression was depleted by RNA interference [22].

Another example of familial cHL involves a whole exome sequencing study with 17 cHL-prone families, which identified a missense mutation from alanine to threonine at the 1065^th^ codon of KDR (also known as VEGFR2) in two independent cHL families [23]. This mutation is located in the activation loop (1046~1075) [24]. It alters a non-polar residue (alanine) into a polar one (threonine) [25], resulting in autophosphorylation on tyrosine even in the absence of vascular endothelial growth factor. Consequently, this mutated residue may impact KDR functions such as tumor angiogenesis and endothelial cell proliferation. Unlike the first case, where the penetrance rate is likely to be close to 1, determining the penetrance rate in missense mutations is crucial for understanding the etiology and pathology of familial cHL. However, obtaining a sufficiently large sample size to estimate statistics for such rare variants accurately poses a challenge.

Alternatively, common genetic variants have been identified in independent individuals. The pathological roles of these variants, identified in sporadic cHL, are often not well understood within the context of the disease. Unlike the clear disruption or constitutional changes observed in familial cHL, variants found in sporadic cHL studies are typically located in intergenic or intronic regions. Mechanisms elucidating gene expression and subsequent regulations are necessary, as demonstrated in a recent multi-OMICS study [26]. This study identified the target genes ERAP1 and ALDH8A1, along with their regulatory nucleotide variants rs27524 and rs6930223, offering plausible scenarios regarding susceptibility to cHL (Table **1**).

The rs27524 variant within a cHL GWAS signal was colocalized with the eGene ERAP1. The minor allele of this variant may enhance the transcriptional activity of ERAP1 by regulating BCL3 and NF-κB. This regulation could lead to an undesirable imbalance between ERAP1 and ERAP2, resulting in weakened HLA class I activity. The insufficient supply of tumor-associated peptide antigens for HLA class I subsequently reduces antigen presentation in HRS cells and hampers their recognition by cytotoxic T cells. This immune escape mechanism contributes to the tumor microenvironment.

The other variant, rs6930223, is located in a linkage disequilibrium block with rs7745098, identified as both a cHL GWAS signal and an eQTL of the eGene ALDH8A1. The minor allele of rs6930223, functioning as an enhancer variant of ALDH8A1, may increase its oxidization activity to the precursor of acetyl-CoA, producing ATP. This process aids in evading apoptosis of pre-apoptotic germinal center B cells. Consequently, the increased production of HRS due to cytokinesis failure raises susceptibility to cHL.

Despite these plausible scenarios, the pathological roles of these functional variants and genes necessitate experimental studies. While recent GWAS efforts have identified 126 signals uploaded to the GWAS Catalog as of November 24, 2023, functional variants and genes are limited, as depicted within the pre-apoptotic cell in Fig. (**1**). These variants may affect gene expression function, but specific scenarios regarding the subsequent pathological mechanisms of cHL have not been suggested thus far.

Before a comprehensive blueprint for the genetic architecture of cHL is available, caution is advised when making predictions about the full spectrum. The genes displayed in the pre-apoptotic cell, as shown in Fig. (**1**), differ significantly from the aberrant genes found in the HRS cell. This difference may be due to the limited representation of genes in the pre-apoptotic cell, potentially representing just the tip of the iceberg compared to the actual polygenic profile increasing susceptibility to cHL. While genetic abnormalities provide a profile defining cHL, susceptibility genes in the pre-apoptotic cell appear to extend to more general driver genes capable of causing cancer rather than being restricted to specific cancers. The multitude of genetic abnormalities dysregulating HRS cell growth can be attributed to original genetic variants under adverse environmental exposures. The original genetic variants regulating susceptibility genes identified by comparing cases to controls play roles in the development and progression of cHL. This is plausible considering the functions of the proto-oncogenes, encompassing metabolism, cytoskeleton, cell cycle, apoptosis, gene regulation, and signaling, as shown in Fig. (**1**). Dysregulation of these functions might contribute to a tumor microenvironment with oncogenic effects. TNFRSF10C, an antagonistic receptor protecting cells from TRAIL-induced apoptosis, is an example, and its genomic deletion and promoter hypermethylation have been found in a variety of cancers, including pancreatic [27], prostate [28], colon [29], lung [30], breast [31], bladder [32] cancers. Serum APOC2, an apolipoprotein member, was proposed as a potential prognostic biomarker for pancreaticoduodenectomy and radiotherapy, respectively, in patients with pancreatic and cervical cancers, showing their significant survivals (*P* < 0.05) by the Kaplan–Meier method [33, 34].

To date, cHL is a relatively precisely defined subtype of cancer, and the outlines of its characteristics reflected from aberrant genes are being revealed. In contrast, explaining the genetic etiology of cHL is limited due to the complexity of polygenic factors. Moreover, genetic aberration is usually confined to tumor cells derived from end-stage cHL patients, making it more difficult to distinguish driver genes from passenger genes. Even taking these into account, our knowledge of genetic risk factors for cHL is thought to be significantly insufficient compared to that of other cancers. For instance, the GWAS Catalog, the most comprehensive platform for human GWAS, shows 126 genetic associations from 16 studies as of November 24, 2023, which is considerably smaller than those for other cancers, including non-Hodgkin lymphoma for which 421 genetic associations were reported from 99 studies. A large volume of further studies, particularly GWAS studies, is inevitable in coping with the era of precision oncology when the current medical paradigm is changing from diagnosis/treatment-oriented medicine to prevention-oriented medicine. Nonetheless, further studies elucidating the landscape of genetic lesions in HRS are warranted to exploit the pathobiology and targeted treatment of cHL as well as their potential as candidate driver genes for genetic variation-based susceptibility to cHL. In particular, further studies are needed to expand our knowledge of the interaction of some genetic lesions in HRS with the tumor microenvironment, which has recently been shown to suppress immunity and promote tumors.

Together with increasing GWAS, multi-OMICS data are required to understand the functional roles of nucleotide sequences at GWAS signals. This is essential to alleviate the unrealistic burden of examining the aberration and expression of genes in the multi-stage progression of cHL, especially from a healthy state to the disease onset or an early stage displaying mononuclear Hodgkin cells.

## 4 STRATEGIES AND PROSPECTS

The resolution of the genetic architecture of cHL susceptibility is currently insufficient to determine clear research directions. Nonetheless, it is critical to adopt more targeted strategies, as shown in Fig. (**2**). In particular, despite heterogeneity in the incidence of cHL by gender, with a higher proportion in males (*e.g*., 85% of the pediatric population [35]), knowledge of gender-specific genetics is quite limited. The higher risk for cHL in sisters (9.4-fold compared to the general population) compared to brothers (4.5-fold) or opposite-sex siblings (5.9-fold) in a previous study [3] strongly advocates in-depth genetic studies of potential sex-specific genetic heterogeneity in cHL.

Another crucial strategy for achieving a higher resolution of susceptibility genes is to perform eQTL mapping and colocalization at all stages of gene expression. Colocalizing loci of different eQTL types (*e.g*., sQTL - splicing eQTL, pQTL - protein eQTL [36, 37]) with cHL GWAS signals will not only help identify more target genes for cHL susceptibility but also allow inferring the context-specific regulatory functions of eQTL. Moreover, an emphasis on single- cell approaches in the genetics of cHL is vital to investigating rare HRS tumor cells and their interactions with diverse non-malignant microenvironments. The cell state-specific effects from this single-cell analysis will help us better understand the progression of HRS cells, as shown in a recent single-cell eQTL study where the allelic effects of 333 eQTL on the corresponding eGenes changed during B cell differentiation [38]. The phenotype-specific genetic architecture contributes to our understanding of the pathophysiology of cHL. For instance, EBV-positive cHL was associated with HLA class I, which may modify cytotoxic lymphocytes (CD8+) response to EBV [39, 40], while EBV-negative cHL was associated with HLA class II [41, 42]. Efforts are needed to elucidate the largely unknown causal relationships between genotypes and other phenotypes of cHL. In the future, researchers will attempt to distinguish cHL genetic architecture by histological subtypes, namely, nodular sclerosis Hodgkin lymphoma, lymphocyte-rich Hodgkin lymphoma, mixed cellularity Hodgkin lymphoma, and lymphocyte-depleted Hodgkin lymphoma, with larger sample sizes.

In the pursuit of identifying and applying genetic factors for cHL, a complex disease, the importance of data analysis using rational methods is underscored. For example, employing mixed models with a genomic similarity matrix helps control polygenetic covariance among individuals and consequently reduces spurious genetic associations [43]. This also addresses concerns about population stratification produced by the admixture of individuals with genetically different backgrounds within and between populations [44]. Genetic heterogeneity by gender can be further implemented in the mixed model analysis by treating men and women data as two different diseases [45, 46]. Moreover, Bayesian inference for mixed model-based genetic associations provides empirical Bayes estimates of genetic effects to avoid violating the assumption of known polygenic and residual variance components required for the best linear unbiased estimator in the frequentist mixed model framework [47, 48]. Moreover, Bayesian inference, which relies on posterior probability estimation, presents numerous benefits in identifying genetic factors contributing to susceptibility to cHL. Analyzing particular phenotypes or subtypes for cHL involves dealing with smaller sample sizes, rendering the application of a frequentist approach challenging. This issue can be addressed by elucidating uncertainty in estimating genetic effects through posterior probability distributions [47]. These distributions reflect prior knowledge, making Bayesian inference increasingly favored as accumulated knowledge on the genetic architecture of cHL grows. The use of Gibbs sampling as a Markov chain Monte Carlo, a numerical algorithm for integrals, enables us to marginalize joint posterior distribution in the Bayesian analysis. For details regarding the advantages of Bayesian inference and the application of Gibbs sampling, refer to [47]. The deep neural network also raises a lot of expectations as a niche method that is hard to utilize within a statistical framework in identifying genetic factors for cHL. However, caution is warranted in interpreting results because of the black-box nature of deep neural networks, lacking statistical properties [37, 49].

Finally, we cannot overstate the importance of functionally validating genetic factors. Omics data analysis often relies on associations, such as GWAS, and even approaches like Mendelian randomization, which can offer causal relationships, require strict assumptions [50]. Thus, there is a chance of identifying a spurious genetic factor for cHL due to the complexity arising from various genetic phenomena, including polygenic inheritance, linkage, epistasis, and pleiotropy. For instance, linkage aids in identifying association signals with variants in proximity to functional variants but hinders the identification of functional variants within the signals [37]. In severe cases, multiple functional variants within a linkage disequilibrium block can lead to antagonistic effects between transcription and translation processes, as shown for the MRPL43 gene [51]. Beyond the reasons mentioned above, another crucial factor emphasizes the need for confirming functionality through experiments. The omics data used for the main analysis or verification often originate from normal individuals, and this does not guarantee that the results obtained from such data will necessarily be applicable to the pathology of cHL. Recent research demonstrates significant advancements in non-invasive methods for profiling and monitoring cHL, which can help address the obstacles of limited malignant HRS cells and the difficulty of applying general population data to cHL pathology [52]. This study revealed that circulating tumor DNA in plasma can surpass representation in bulk tumors, facilitating non-invasive profiling.

## CONCLUSION

Recent research has advanced our comprehension of cHL. Specifically, numerous genes identified through genetic aberrations in cHL have been implicated in distinct pathways involved in the progression of the disease. Nevertheless, there appears to be a notable gap in understanding the driver genes and their nucleotide sequence variants underlying the onset and progression of cHL. Vigorous research endeavors aimed at identifying these genetic factors are essential to propel and enhance the era of precision oncology. The proposed strategies in this article will provide valuable insights into the genetic complexity of cHL.

## AUTHORS’ CONTRIBUTIONS

All authors have accepted responsibility for the entire content of this manuscript, consented to its submission to the journal, and approved the final version of the manuscript.

## Figures and Tables

**Fig. (1):**
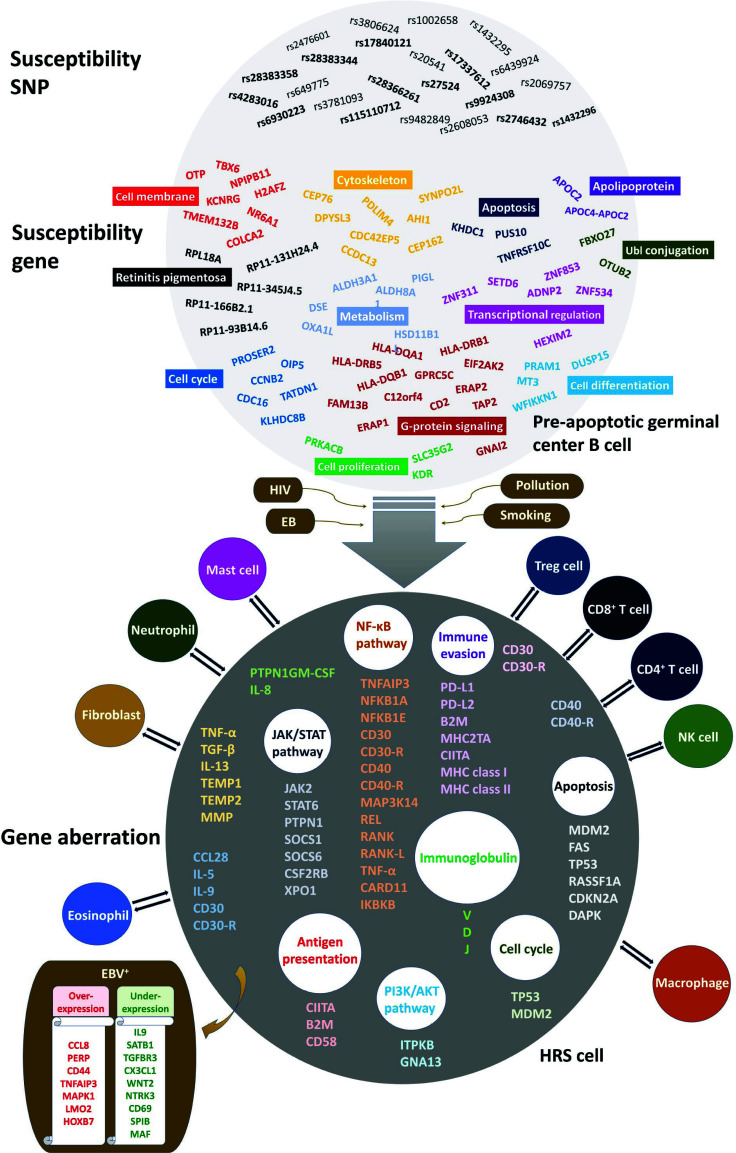
Genetic architecture of classical Hodgkin lymphoma (cHL). Pre-apoptotic cell presents regulatory single uncleotide polymorphisms (SNPs, upper part), and their target genes (eGenes, lower part), and Hodgkin and Reed-Sternberg (HRS) cell presents gene aberration after cHL onset. All the displayed SNPs were colocalized by cHL genomewide association study signal and expression quantitative trait locus (eQTL), and SNPs with regulatory function are in bold. The susceptibility genes were eGenes corresponding to eQTL or identified in at least two families. They are categorized by biological function. Human immunodeficiency virus (HIV), Epstein-Barr virus (EBV), pollution, and smoking are presented as enviromental factors. Genetic lesions are classified by pathways and mechanisms underlying pathophysiology of cHL. The cells outside the HRS cell show cHL microenvironment, including diverse innate and adaptive immune cells. Under EBV exposure (EBV^+^), up- and down-regulated gene profiles [9] are presented as a representative example of interaction between genetic and environmental factors.

**Fig. (2):**
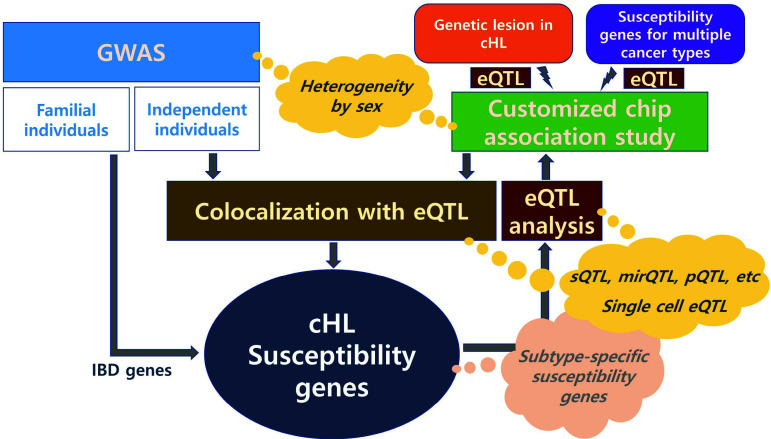
Genetic studies for discovery of classical Hodgkin lymphoma (cHL) susceptibility genes. The speech bubbles refer to strategies that are not currently being implemented but are necessary to increase resolution of genetic architecture of cHL susceptibility. IBD: identical-by-descent.

**Table 1:** Functional nucleotide sequence variant/mutation, functional gene, and pathogenesis for familial and sporadic cHL^a^.

**-**	**Familial cHL**		**Sporadic cHL**	
**Functional nucleotide sequence variant/mutation**				
**ID**	t(2,3) (q11.2;p21.31)	rs56302315	rs27524	rs6930223
**Type**	Reciprocal translocation	SNV	SNV	SNV
**Location^b^**	Chromosome 2, q11.2 ↔ Chromosome 3, p21.31	4:55089802 Exonic variant in KDR	5:96766240 Intronic variant in ERAP1	6:135103065 Intergenic variant
**Function**	Deletion of the 5’UTR and promoter of KLHDC8B	Missense mutation altering an amino acid from alanine to threonine	Promoter Transcription factor (NF-κB, BCL3) binding	Enhancer Activator (AP1) binding
**Associated phenotype**	-	-	Hodgkin lymphoma [19], psoriasis [19]	Hodgkin lymphoma [20]
**Functional gene**				
**Name**	Kelch domain containing 8B	Kinase insert domain receptor	Endoplasmic reticulum aminopeptidase 1	Aldehyde dehydrogenase 8 family member A1
**Abbreviation**	KLHDC8B	KDR	ERAP1	ALDH8A1
**Location^b^**	3:49171598..49176486	4:55078481..55125595	5:96760813..96935854	6:134917393..134950101
**Function**	Forming a beta-propeller protein structure of kelch domains allowing for protein-protein interactions	Tumor angiogenesis, endothelial proliferation, and survival	Aminopeptidase in trimming HLA-binding precursors, often acting as a heterodimer with ERAP2	Catalyze conversion of 2-aminomuconic semialdehyde to 2-aminomuconate in the kynurenine pathway
**Pathogenesis**				
**Risk allele**	NA	T (Mutation allele)	A (Minor allele)	G (Minor allele)
**Gene expression**	Decrease KLHDC8B	NA	Decrease ERAP1, Imbalance between ERAP1 and ERAP2	Increase ALDH8A1
**Dysfunction**	Impaired cytokinesis	Gain of function promoting constitutive autophosphorylation of KDR	Reduced supply of tumor-associated antigens to HLA	Undesirable cell survival with more ATP by OXPHOS
**Induction into HRS**	Increase binucleated cells, producing HRS	Clonal selection of HRS	Immune escape by failed recognition of HRS	HRS produced by evading apoptosis
**References**	[22]	[23]	[26]	[26]

**Note: **^a^This table presents representative functional nucleotide sequence variants/mutations that may affect susceptibility to familial and sporadic lymphoma. ^b^Chromosomal location is presented according to the human reference genome assembly GRCh38.

## LIST OF ABBREVIATIONS

cHL: Classical Hodgkin lymphoma
eQTL: Expression quantitative trait locus
GWAS: Genome-wide association studies
HRS: Hodgkin and Reed-Sternberg
JAK/STAT: Janus kinase/signal transducer and activator of transcription
NF-kB: Nuclear factor kappa B
PI3K/AKT: Phosphoinositide 3-kinase/protein kinase B
